# Supplementary material for: Dynamic regulation of murine RNA polymerase III transcription during heat shock stress
Source: Genetics. 2025 Mar 18;230(1):iyaf042. doi: 10.1093/genetics/iyaf042 (PMC12059648; doi:10.1093/genetics/iyaf042)
Supplement: iyaf042_Supplementary_Data [file iyaf042_supplementary_data.zip › Supplemental_Figure_Legends_GENETICS-2025-307891.docx]

**Figure S1. Replicate analysis of Pol III CUT&Tag in wildtype mESCs and PRO-seq in wildtype MEFs. (A)** Genome-wide average plots (top) and heatmaps (bottom) arranged by decreasing signal for Pol III CUT&Tag (maroon) in wildtype mESCs and PRO-seq in wildtype MEFs (green) in a 400 bp window surrounding the TSS of all tRNA genes with two biological replicates.

**Figure S2. tRNA transcription is impacted upon heat shock treatment in wildtype mESCs. (A)** Two biological replicates of immunoblot analysis (left) and relative quantification (right) with α-RPC7 (Pol III), normalized to α-Tubulin, of whole cell extracts from wildtype (WT) mESCs treated with 0 minutes (HS0), 30 minutes (HS30), and 60 minutes (HS60) of heat shock. Statistical analysis was performed using one-way ANOVA. ns: non-significant. **(B)** Gene browser tracks at *tRNA71-AlaAGC* (top) and *tRNA1040-ArgACG, tRNA27-TyrGTA, tRNA28-TyrGTA* (bottom) of reads from NET-seq in HS0 and HS30-treated wildtype mESCs. **(C)** Genome-wide average plots (top) and heatmaps (bottom) arranged by decreasing signal for NET-seq in wildtype mESCs in a 50 bp window surrounding the TSS of all tRNA genes with two biological replicates (left). Individual biological replicates are shown on the right. **(D)** Normalized read counts of NET-seq signal in HS0 vs. HS30-treated wildtype mESCs from the TSS to the TES of all tRNA genes. **(E)** Schematic for the enrichment of BrU-labeled RNA for BRI-qPCR in HS0, HS30, and HS60-treated mESCs. **(F)** RT-qPCR analysis of *tRNA71-AlaAGC*, *tRNA27-TyrGTA,* and *Hspa1a* normalized to NLuc signal (n=2, mean ± SD) in wildtype mESCs treated with MG132 for 0 minutes (blue), 120 minutes (red), and 240 minutes (grey). Statistical analysis was performed using one-way ANOVA. ns: non-significant. **: p≤ 0.01. ***: p ≤ 0.001.

**Figure S3. Replicate analysis of Pol III CUT&Tag on tRNAs in *Hsf1*^-/-^ mESCs.** **(A)** RT-qPCR analysis of *Hspa1a* normalized to NLuc signal (n=2, mean ± SD) in wildtype (left) and *Hsf1*^-/-^ (right) mESCs treated with 0 minutes (HS0), 30 minutes (HS30), and 60 minutes (HS60) of heat shock. Statistical analysis was performed using one-way ANOVA. ns: non-significant. ***: p ≤ 0.001. **(B)** Genome-wide average plots (top) and heatmaps (bottom) arranged by decreasing signal for Pol III CUT&Tag (maroon) in *Hsf1*^-/-^ mESCs in a 400 bp window surrounding the TSS of all tRNA genes with two biological replicates. **(C)** Immunoblot analysis (top) and relative quantification (bottom) with α-RPC7 (Pol III), normalized to α-Tubulin of HS0-treated, HS30-treated, and HS60-treated *Hsf1*^-/-^ whole cell extracts. Statistical analysis was performed using one-way ANOVA. ns: non-significant.

**Figure S4. Replicate analysis of Pol III CUT&Tag on Pol III-transcribed ncRNAs in wildtype and *Hsf1*^-/-^ mESCs. (A)** Gene browser tracks at *n-R5s136* (left), *Rn7s1* (middle), and *Rn7sk* (right) of reads from Pol III CUT&Tag in wildtype (WT) and *Hsf1*^-/-^ mESCs with 0 minutes (HS0), 30 minutes (HS30), and 60 minutes (HS60) of heat shock with two biological replicates.

**Figure S5. Replicate analysis of Pol III CUT&Tag on tRNAs in preconditioned wildtype and *Hsf1*^-/-^ mESCs.** **(A-B)** Genome-wide average plots (top) and heatmaps (bottom) arranged by decreasing signal for Pol III CUT&Tag (maroon) in preconditioned wildtype (WT) (A) and preconditioned *Hsf1*^-/-^ (B) mESCs treated with 0, 10, 30 or 60 minutes of heat shock (PHS0, PHS10, PHS30, PHS60, respectively) in a 400 bp window surrounding the TSS of all tRNA genes with two biological replicates.
