# Supplementary figures and images for: Dynamic regulation of murine RNA polymerase III transcription during heat shock stress
Source: Genetics. 2025 Mar 18;230(1):iyaf042. doi: 10.1093/genetics/iyaf042 (PMC12059648; doi:10.1093/genetics/iyaf042)

**A**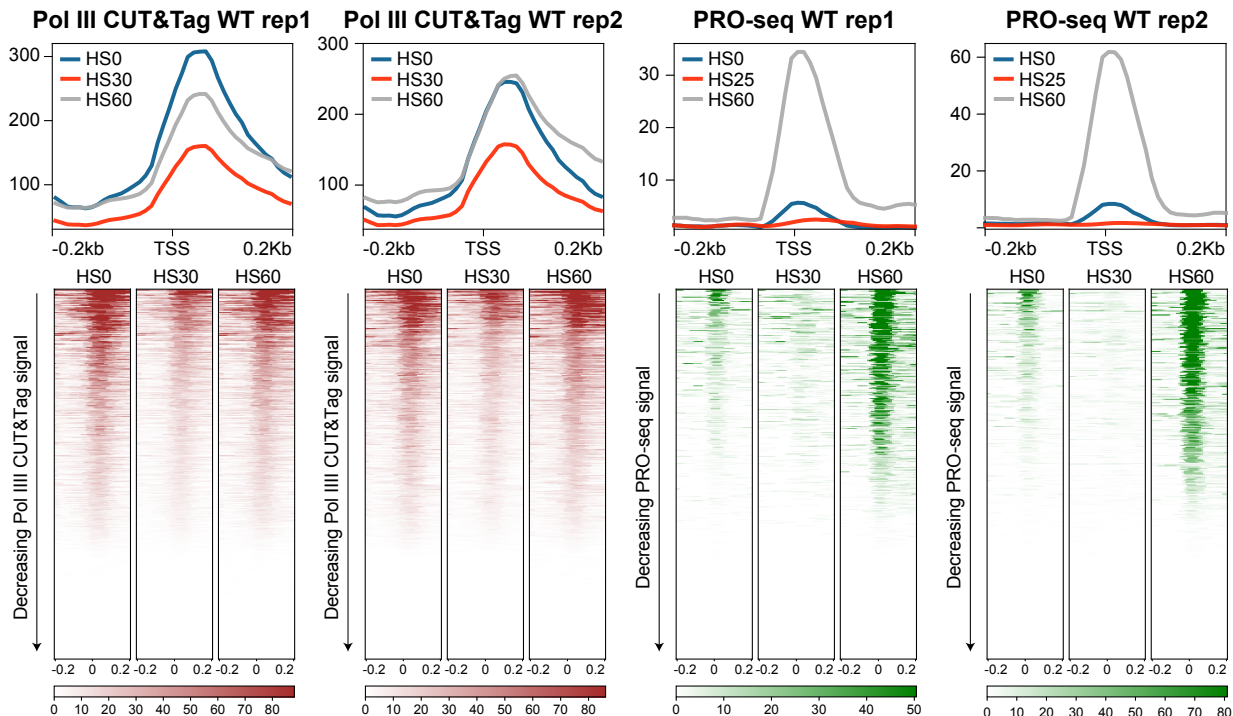**Figure S1**

Supplement: iyaf042_Supplementary_Data [file iyaf042_supplementary_data.zip › Figure_S1_GENETICS-2025-307891.pdf]

**A**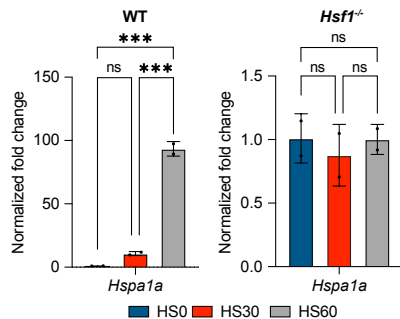**B**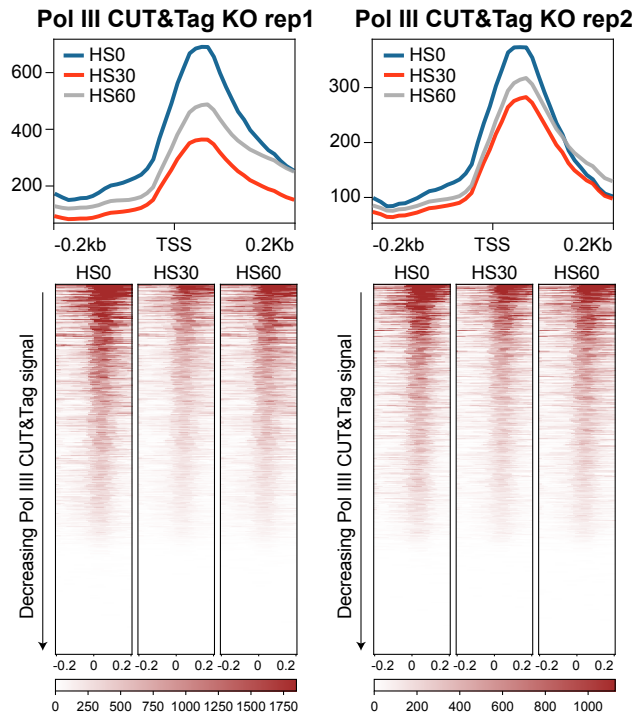**C**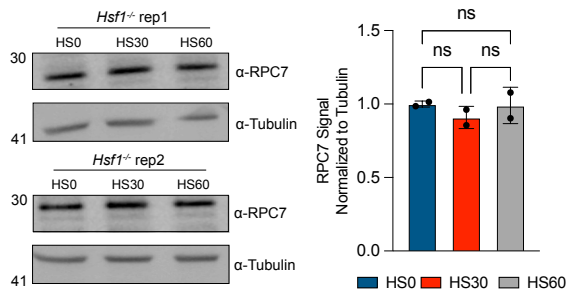**Figure S3**

Supplement: iyaf042_Supplementary_Data [file iyaf042_supplementary_data.zip › Figure_S3_GENETICS-2025-307891.pdf]

**A**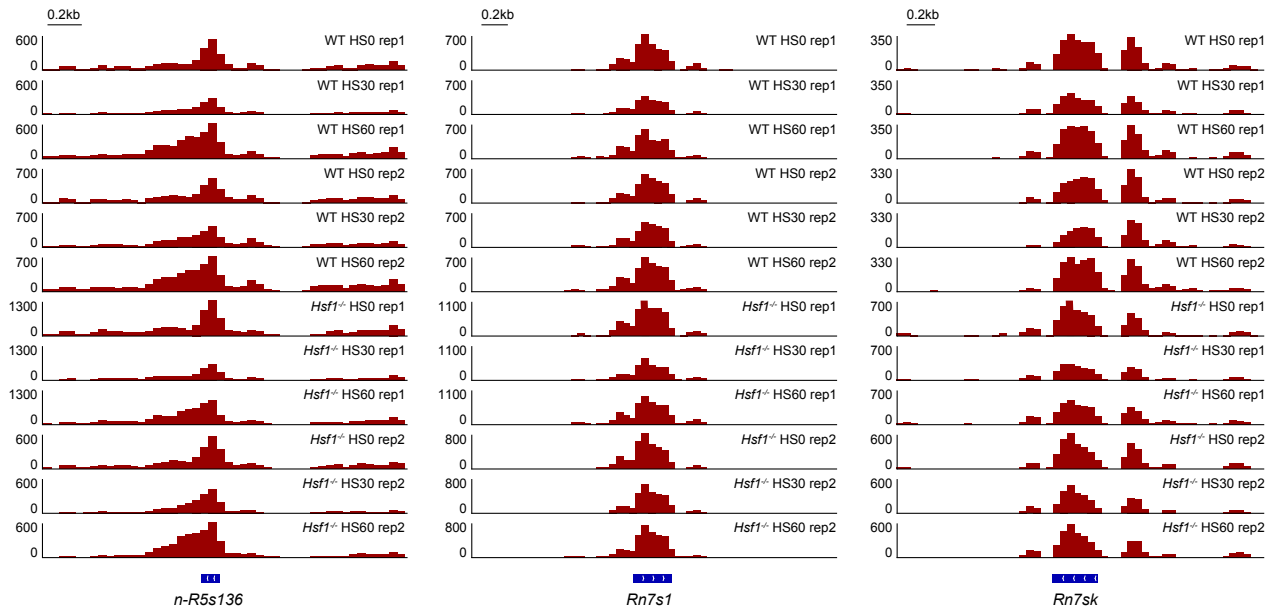**Figure S4**

Supplement: iyaf042_Supplementary_Data [file iyaf042_supplementary_data.zip › Figure_S4_GENETICS-2025-307891.pdf]

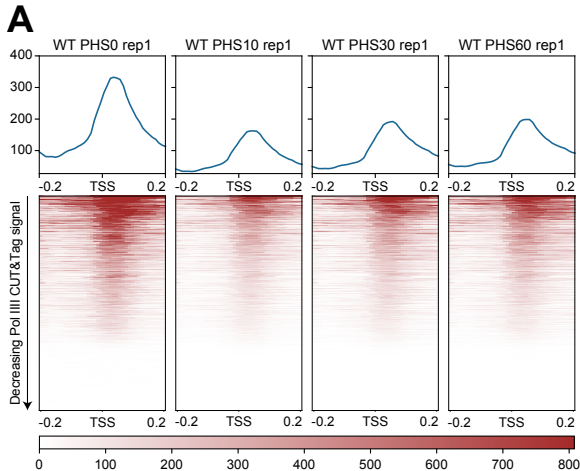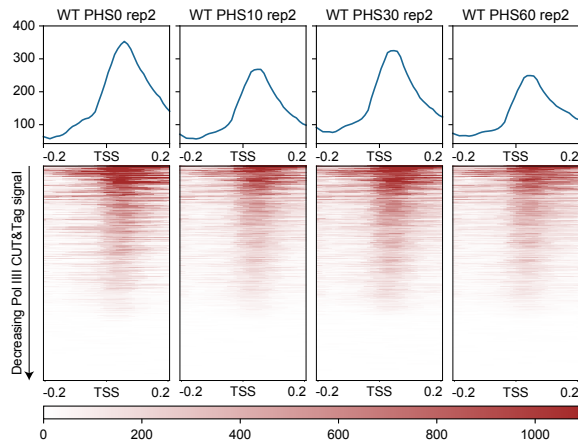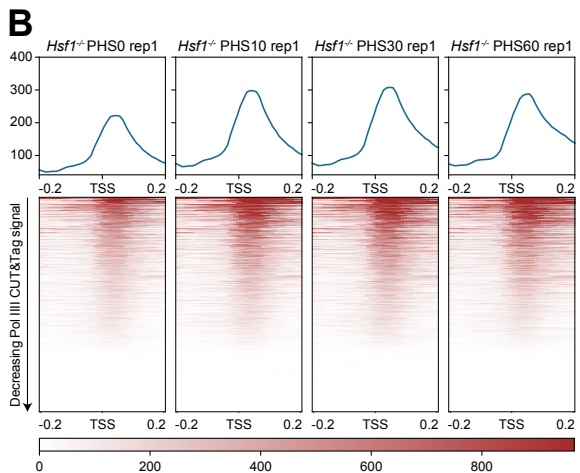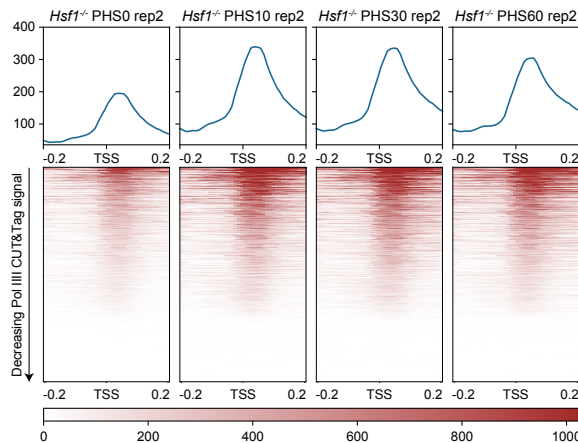

**Figure S5**

Supplement: iyaf042_Supplementary_Data [file iyaf042_supplementary_data.zip › Figure_S5_GENETICS-2025-307891.pdf]
