# Supplementary material for: Dynamic regulation of murine RNA polymerase III transcription during heat shock stress
Source: Genetics. 2025 Mar 18;230(1):iyaf042. doi: 10.1093/genetics/iyaf042 (PMC12059648; doi:10.1093/genetics/iyaf042)
Supplement: iyaf042_Supplementary_Data [file Figure_S2_GENETICS-2025-307891.pdf › Figure_S2_GENETICS-2025-307891.pdf]

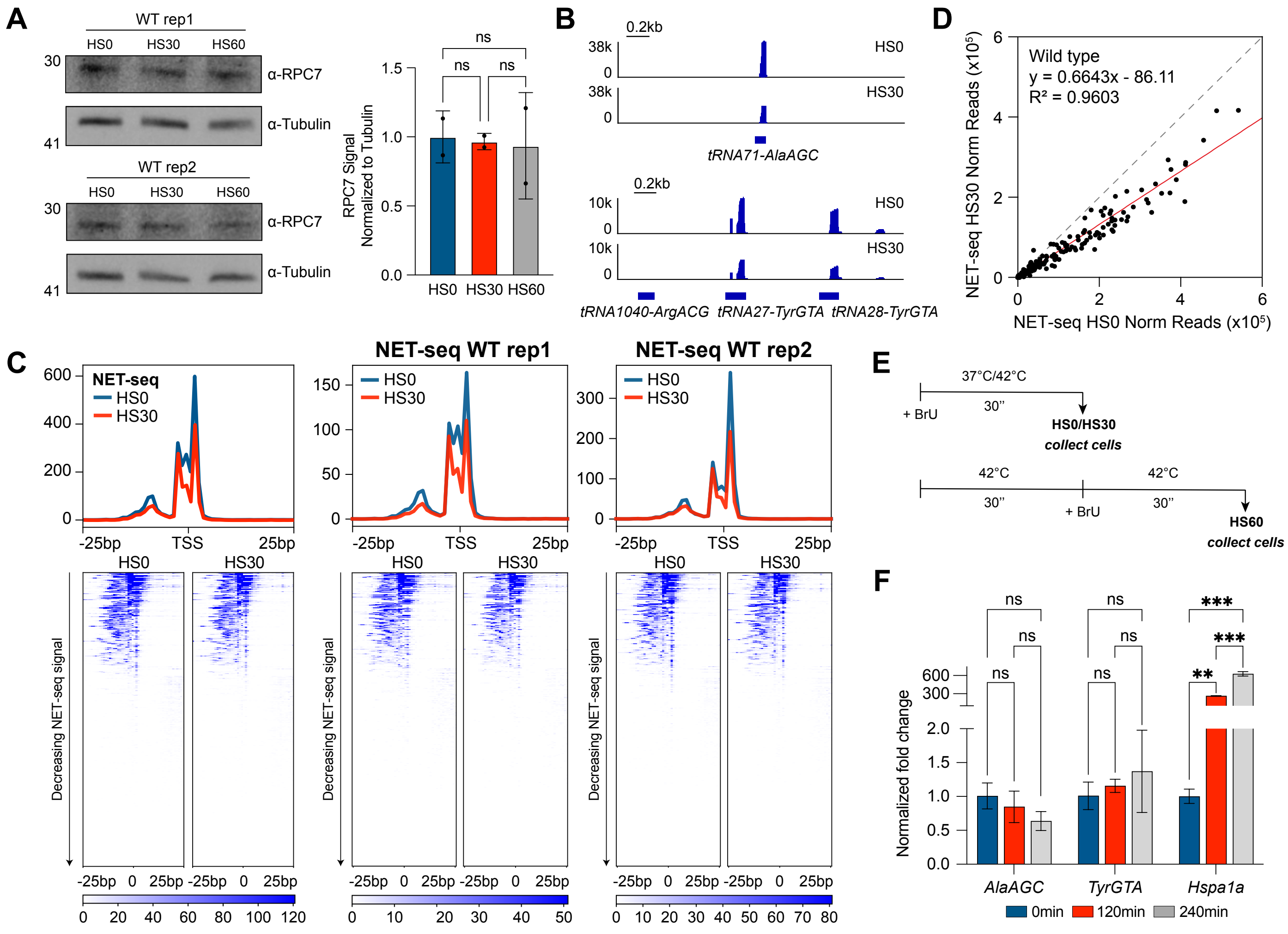
A
WT rep1
HS0 HS30 HS60
30
α-RPC7
α-Tubulin
41
WT rep2
HS0 HS30 HS60
30
α-RPC7
α-Tubulin
41
ns
ns ns
RPC7 Signal Normalized to Tubulin
1.5
1.0
0.5
0.0
HS0 HS30 HS60
B
0.2kb
38k
0
HS0
38k
0
HS30
tRNA71-AlaAGC
0.2kb
10k
0
HS0
10k
0
HS30
tRNA1040-ArgACG tRNA27-TyrGTA tRNA28-TyrGTA
D
Wild type
y = 0.6643x - 86.11
R² = 0.9603
NET-seq HS30 Norm Reads (x10⁵)
NET-seq HS0 Norm Reads (x10⁵)
0 2 4 6
C
NET-seq
HS0
HS30
600
400
200
0
-25bp TSS 25bp
NET-seq WT rep1
HS0
HS30
150
100
50
0
-25bp TSS 25bp
NET-seq WT rep2
HS0
HS30
300
200
100
0
-25bp TSS 25bp
HS0 HS30
Decreasing NET-seq signal
-25bp 0 25bp -25bp 0 25bp
0 20 40 60 80 100 120
0 10 20 30 40 50
0 10 20 30 40 50 60 70 80
E
37°C/42°C
+ BrU
30''
HS0/HS30
collect cells
42°C
42°C
30''
+ BrU
30''
HS60
collect cells
F
ns
ns
ns
ns
ns
ns
***
***
**
Normalized fold change
600
300
2.0
1.5
1.0
0.5
0.0
AlaAGC TyrGTA Hspa1a
0min 120min 240min

Figure S2
